# Supplementary material for: Diversification of biofilm architecture among freshwater Pararheinheimera isolates
Source: Appl Environ Microbiol. 2026 Aug 28;92(9):e00400-26. doi: 10.1128/aem.00400-26 (PMC13599514; doi:10.1128/aem.00400-26)
Supplement: Supplementary file 1 [file aem.00400-26-s0001.docx]

**Supplemental Material**

Diversification of biofilm architecture among freshwater *Pararheinheimera* isolates

Amanda E. Appel^1^, Alexandra G. Goetsch^1^, Ellen W. van Wijngaarden^2,3^, Daniel J. Novacek^1^, Peter M. Burnham^1^, Meredith N. Silberstein^2,3^, and David M. Hershey^1^ #

^1^ Department of Bacteriology, University of Wisconsin – Madison, Madison, WI, USA

^2^ Sibley School of Mechanical and Aerospace Engineering, Cornell University, Ithaca, NY, USA

^3^ Engineered Living Materials Institute, Cornell University, Ithaca, NY, USA

Running title: Biofilm formation in *Pararheinheimera sp.*

\# To whom correspondence should be addressed: dhershey@wisc.edu

**Figure S1:** Statistical analysis of crystal violet staining. *Pararheinheimera* isolates and reference strain cultures analogous to those shown in Fig. 3B were crystal violet stained as described in Materials and Methods. Statistical significance was evaluated using a one-way ANOVA followed by Tukey’s HSD post-hoc test. Strains B2H12, DN11 and DN20 are indistinguishable from the medium-only control. B1H7 exhibits weak staining, and B2H7 shows moderate staining relative to adherent strains. Error bars represent the standard deviation of four biological replicates. Letters denote statistically significant differences (p < 0.05).

**Figure S2:** Properties of VM-matrices are consistent across independent extractions. EPS was extracted from three independent cultures of strain B2H12 (blue) and B1H7 (magenta) as described in Materials and Methods. For the strain and frequency sweeps, filled markers represent the storage modulus (G’) and unfilled markers represent the loss modulus (G’’). Each extract was dissolved in water at 1% (w/v) and subjected to bulk rheology testing. Extracts consistently displayed shear-thinning behavior and formed hydrogels. Matrix from B2H12 consistently displayed dynamic moduli that were an order of magnitude higher than those from B1H7.

**Figure S3:** Representatives from all five *Pararheinheimera* subclades are motile in soft agar. Strains were grown to saturation in liquid PYE at room temperature, and 1uLof these starter cultures was inoculated into PYE medium solidified with 0.25% (w/v). Plates were incubated at room temperature and imaged at the times indicated.
